# Rolf Jessberger: cohesin, telomeres, & germ cells

**DOI:** 10.26508/lsa.202302208

**Published:** 2023-06-22

**Authors:** Rolf Jessberger

**Affiliations:** Institute of Physiological Chemistry, Medical Faculty Carl Gustav Carus, Technische Universität Dresden , Dresden, Germany

## Abstract

Rolf Jessberger is Professor and Chairman at the Institute of Physiological Chemistry, and Faculty of Medicine at the Technische Universität Dresden. We asked him about his recent article published in Life Science Alliance (LSA) and his experience in science thus far.

## Abstract (precis)

Interview with Rolf Jessberger

**Figure 1:**
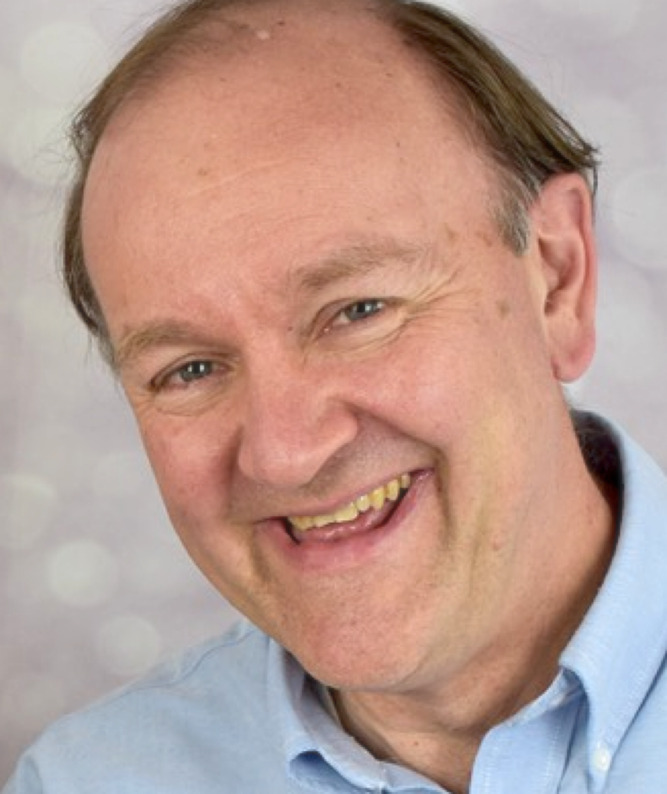
Rolf Jessberger

## How would you explain the main findings of your article, and how did it come about?

This article deals with germ cells, more precisely with meiocytes and the integrity of their chromosomes. For higher organisms, germ cells may be considered the “origin of all individual life,” therefore the faithful transmission of intact chromosomes is essential. Cohesins, best known for their roles in sister chromatid cohesion, and topological control of chromosomes, and thus regulation of gene expression, are key to chromosome structure and dynamics not only in somatic cells, but certainly also in germ cells. In spermatocytes and oocytes, several distinct cohesin complexes exist, some of them composed of meiosis-specific subunits. One such meiotic subunit is SMC1β, identified by us many years back (Revenkova et al, 2001), which replaces the canonical SMC1α present in somatic cells and at early stages in meiosis, in most of the meiotic cohesin complexes. Roles of SMC1β were described by us in sister chromatid cohesion up to metaphase II of meiosis in determining chromosome axes length, and thereby, the axis to loop ratio, in supporting double-strand break repair and meiotic recombination, and in protecting telomers of spermatocytes and oocyte chromosomes (Revenkova et al, 2004; Hodges et al, 2005; Novak et al, 2008; Adelfalk et al, 2009; Revenkova et al, 2010). The question emerged: why are there two SMC1 variants in vertebrate meiocytes, and is there some function that only SMC1β can fulfill? To address this question, we expressed SMC1α under the control of the SMC1β promoter region in SMC1β-deficient mice and analyzed the rescue of SMC1β-deficiency phenotypes by SMC1α (Biswas et al, 2018). It turned out that SMC1α can rescue nearly all the meiotic phenotypes observed in SMC1β-deficient mice—except telomere damage. Thus, here is an SMC1β-specific role. How does SMC1β control telomere integrity? Telomerase expression and expression and localization of shelterin proteins appeared normal in SMC1β-deficient meiocytes. What else may depend on SMC1β? How about TERRA, the long noncoding RNA transcribed from subtelomeric/telomeric regions? Too much or too little TERRA was described to be deleterious, causing telomere damage. Does SMC1β regulate TERRA expression? Yes, it does, as became quickly clear by analyzing TERRA expression from a series of chromosomes in spermatocytes (Biswas et al, 2023). Increased TERRA expression may cause telomere damage through TERRA R-loop formation at telomeres. Does this indeed happen in SMC1β-deficient spermatocytes? Using an antibody which detects R-loops, it became clear that there are more R-loops at the chromosome ends of these spermatocytes. Thus, SMC1β governs TERRA expression and thereby, telomeric R-loop formation. How does SMC1β control TERRA expression? Using both ATACSeq, to test for open, accessible chromatin, and RNASeq, to assess transcriptional activity very close to chromosome ends compared with regions of increasing distance to the ends, it turned out that SMC1β promotes closed chromatin and restricts transcription near the ends. Chromatin gets more accessible and more transcribed the further one moves away from the ends. Thus, we are suggesting a mechanism by which SMC1β contributes to maintenance of intact telomeres, that is, through controlling chromatin accessibility and thereby, TERRA expression. Telomere damage through weakening of this control may increase when SMC1β cohesin complexes get lost as is likely happening in ageing oocytes. Besides the previously described process of cohesin decay as a major contributor to age-dependent increase in chromosome mis-segregation and thus aneuploidy (Jessberger, 2012), telomere damage through the above mechanism may also add to problems transmitting intact chromosomes.

## What was the decision process in choosing where to publish?

We had earlier published in *Life Science Alliance* (McNicoll et al, 2020) and found the submission, review, and publication process very straightforward. Thus, when our current article was referred from *The EMBO Journal* to *LSA*, we happily agreed to have the article transferred. The experience with the reviewing and editorial process again was very pleasant. Nowadays, some publishers overload the process with formal requirements that in my opinion—although transparency and data integrity and availability are of course a must—do not increase the value and the quality of the article, but just pose unnecessary burden on the scientists. *LSA* keeps very high standards but limits formalities, which is much appreciated.

## How do you think publishing in an open access journal like Life Science Alliance has impacted the visibility of your findings?

Since several years, we try to publish open access whenever possible. I think this is a great concept and there are many arguments supporting it. Making research results available around the globe, also for those that are not as privileged as we are, is one the most important reasons. Giving science freely back to the public—who largely pay for it through their taxes—is another. The unfortunate behavior of some publishing houses adds to the demand for open access. Many granting agencies have adapted a strategy to support or even demand open access publication, and this is a beneficial trend. These and other arguments are well known, and I strongly believe that visibility of one’s own research is substantially enhanced through open access, such as at *LSA*.

## What advice do you have for other researchers on maximizing the dissemination of their work?

Well, this follows from the above and that advice is probably targeted a bit more to the younger researchers among us: try to publish in open access journals. That is better for your science, better for the science community, and better for everyone everywhere. Besides the science article itself, it is also important to convey key messages, maybe in a much-simplified mode, to the general public such as in public science events or through serious social media, or at whatever small and large opportunities. As science and thus scientific education is the only way out of major problems of society and of our planet, it is important to tell many about science in general and about your work specifically. Every solid contribution counts.

## What questions is your laboratory currently trying to answer?

In the cohesin/germ cell area—my laboratory also works on certain aspects of immunology—we like to address several questions. Such as the one after the current article on TERRA: is there indeed a higher TERRA expression in old oocytes and is this associated with loss of SMC1β? Ageing experiments obviously take time and thus patience is required for such studies. Other experiments focus on the most obvious difference between the otherwise very similar SMC1⍺ and SMC1β proteins: the C-terminus where SMC1β features an additional 21 amino acids, initially characterized by us earlier, is not present in SMC1⍺. Generating a mutant lacking that sequence or having this sequence added to SMC1⍺ are experiments we are executing or preparing for. A question that is so far only partially answered still is about some of the individual functions of the six or so distinct cohesin complexes that meiocytes form. Interacting proteins and the respective processes are also insufficiently known. As there is no bona fide cell culture system for germ cells to faithfully recapitulate meiosis, such experiments require many mouse mutants to study meiosis in vivo and meiocytes ex vivo. This is also true in a related research area in the laboratory focusing on the role of the CENP-V protein, which is scarcely described and was reported by us recently to control the oocyte spindle (Nabi et al, 2021). How this protein mechanistically contributes to oocyte spindle formation is one of the questions we are trying to address, which obviously has implications for basic germ cell biology and reproductive health.

## What motivated you to pursue a career in science, and what have been the most interesting moments on the path that led you to where you are now?

When as a young student, fresh from high school, I decided to go for life science; this was purely driven by curiosity about the living world. All during childhood, I was interested in outdoors and its fascinating nature; both my grandparents were biologists who started their studies and research in the 1920s, and this was the ground laid for my attachment to biology and later to molecular processes. I never really thought much about salary, job safety, retirement plans, vacation days, and all that, but was full of optimism that a life in science and a life for science is the very best, not only for myself, but for contributing at least a teeny tiny bit to the progress of mankind. Sounds too idealistic, naive? Maybe from today’s perspective, but at the bottom of my heart, I still think this is the right attitude. The key to fostering my scientific thinking were my PhD supervisor Prof. Walter Doerfler, a very accomplished molecular cell biologist and virologist, and my postdoc mentor Prof Paul Berg, Nobel laureate at Stanford University’s Department of Biochemistry. Getting exposed to how research works in different countries—for me, these were the in USA twice, Switzerland, and Germany—was also a very valuable lesson.

## Tell us something interesting about yourself that would not be on your CV

I firmly believe that science is the only solution to the major problems that mankind faces, from climate change to infectious diseases, from cancer to hunger, from environmental pollution to preserving ecosystems, etc., etc. Science shall not, however, be just “cold rationalism,” but must be embedded in high standards of humanism, of ethics, and the consequences of new scientific revolutions must be thought about and acted upon. Every scientist should act responsibly and fairly not only within the world of research, that is, to laboratory members, colleagues, and partners, but generally—despite all the pressures that many scientists are experiencing such as competition, funding. This kind of credo—put here very briefly and thus a bit superficially—would not be found in a typical CV but is important to me.
